# The Lipid Parameters and Lipoprotein(a) Excess in Hashimoto Thyroiditis

**DOI:** 10.1155/2015/952729

**Published:** 2015-05-03

**Authors:** D. O. Yetkin, B. Dogantekin

**Affiliations:** ^1^Department of Endocrinology, Kolan International Hospital, 36384 Istanbul, Turkey; ^2^Department of Internal Medicine, Goztepe Education and Research Hospital, 34722 Istanbul, Turkey

## Abstract

*Objective*. The risk of atherosclerotic heart disease is increased in autoimmune thyroiditis, although the reason is not clear. Lipoprotein(a) (Lp(a)) excess has been identified as a powerful predictor of premature atherosclerotic vascular diseases. The aim of this study is to investigate the relationship between Lp(a) levels and thyroid hormones in Hashimoto patients. *Method*. 154 premenopausal female Hashimoto patients (48 patients with overthypothyroid (OH), 50 patients with subclinical hypothyroid (SH), and 56 patients with euthyroid Hashimoto to (EH)) were enrolled in this study. The control group consists of 50 age matched volunteers. In every group, thyroid function tests and lipid parameters with Lp(a) were measured. Lp(a) excess was defined as Lp(a) > 30 mg/dL. *Results*. Total-C, LDL-C, TG, and Lp(a) levels were increased in Hashimoto group. Total-C, LDL-C, and TG levels were higher in SH group than in the control group. Total-C and LDL-C levels were also higher in EH group compared to controls. Lp(a) levels were similar in SH and EH groups with controls. However, excess Lp(a) was more common in subclinical hypothyroid and euthyroid Hashimoto group than in the control group. *Conclusion*. The Total-C and LDL-C levels and excess Lp(a) were higher even in euthyroid Hashimoto patients. Thyroid autoimmunity may have some effect on Lp(a) and lipid metabolism.

## 1. Introduction

Thyroid hormones regulate the wide array of metabolic parameters [1]. They have significant effect on the synthesis, mobilization, and metabolism of lipids and lipoproteins [2]. Dyslipidemia is a common metabolic abnormality in patients with thyroid disease, in either the overt or the subclinical forms of the disease. It has been known that overt hypothyroidism is associated with premature coronary heart disease and one of the components of this assumed to be a deteriorated metabolism of atherogenic lipoproteins. Lipoprotein(a) (Lp(a)) is a very large protein molecule wrapped around, and linked by a disulphide bond to, a LDL-like particle. There is a strong relationship with Lp(a) and coronary heart disease, independent of the standard vascular risk factors [3]. Autoimmune thyroiditis (Hashimoto thyroiditis) is a very common disorder and it is the main reason for hypothyroidism [4]. There are considerable sufficient data between the overt hypothyroidism, dyslipidemia, and coronary heart disease, although there are conflicting reports on whether subclinical hypothyroidism or euthyroid autoimmune thyroid disease has any influence on the lipoprotein metabolism. Recently it was reported that thyroid autoimmunity may be an important mechanism for the occurrence of atherosclerosis [5] and it is well known that Lp(a) is one of the main risk factors for the atherosclerosis.

The aim of this study was to investigate the relationship of lipid parameters, Lp(a) levels, and thyroid hormones in Hashimoto thyroiditis patients.

## 2. Patients and Methods

One hundred and fifty-four premenopausal female Hashimoto patients with a mean age of 37.19 ± 7.98 SD years, followed up and treated in our outpatient clinic between July 2013 and October 2013, were enrolled in this study. Forty-eight patients had overt hypothyroidism (age: 35.79 ± 7.29 SD years), fifty patients had subclinical hypothyroidism (age: 36.8 ± 8.19 SD years), and fifty-six patients were euthyroid (36.3 ± 8.11 SD years). All patients were positive for both antithyroid peroxidase and antithyroglobulin autoantibodies. The control group consisted of 50 healthy volunteers with a mean age of 35.42 ± 7.64 SD years.

The diagnosis of overt hypothyroidism (OH) was based on clinical findings, low serum free thyroxine (FT4) levels, and high serum thyroid stimulating hormone (TSH) concentrations. Subclinical hypothyroidism (SH) was established in terms of TSH levels ≥ 4.2 mIU/mL and normal FT4 levels. Euthyroid Hashimoto (EH) was established as normal FT4 and TSH levels with only positive thyroid autoantibodies.

Patients and controls with diabetes mellitus, current and ex-smokers, obesity (body mass index (BMI) > 30 kg/m²), liver disease and systemic illness, excessive alcohol consumption, and a known family history of primary hyperlipidemia were excluded from the study. None of the subjects were receiving treatment with estroprogestin therapy, diuretics, ß blockers or lipid lowering drugs, or other medications that might alter serum lipid parameters and thyroid functions. None of the patients were receiving thyroid treatment and lipid level determinations were done on initial presentation, before the treatment.

The simple index to determine insulin resistance [i.e., homeostasis model assessment (HOMA-R) > or = 2.5] was used [6], and subjects who were determined to be positive for insulin resistance according to this index were also excluded. The study was approved by local ethical committee. Written informed consent was obtained from all the subjects.

Blood samples were collected after an overnight fast from an antecubital vein. Chemiluminescence immunoassay was done to assess TSH (normal 0.4 to 4.2 mIU/mL), free thyroxine (normal: 0.93 and 1.7 ng/dL), free triiodotironin (normal: 2.5–4.3 ng/mL), and serum autoantibodies against thyroglobulin (TgAb) (normal: 0–115 IU/mL) and thyroid peroxidase (TPOAb) (normal: 0–34 IU/mL ECLIA (Modular Analytics E170; Roche Diagnostics)). The measurements of total cholesterol (Total-C) (normal: 130–200 mg/dL), high density lipoprotein (HDL-C) (normal: >50 mg/dL), low density lipoprotein (LDL-C) (normal: 70–130 mg/dL), and triglyceride (TG) (normal: 60–150 mg/dL) levels were determined by enzymatic methods. Lipoprotein(a) (normal 0–30 mg/dL) was measured in turbidimetric method by spectrophotometry (CRONY, JOOLY 100). Lp(a) excess was defined as Lp(a) > 30 mg/dL [7].

Data were analyzed by SPSS 14 (SPSS Inc., Chicago, IL, USA) software. Normality of the distribution of variables was controlled with Kolmogorov-Smirnov test. Continuous data were expressed as the mean ± SD and median and interquartile ranges, when appropriate. One-way ANOVA, Mann-Whitney *U*, and Kruskal Wallis tests were used to compare the parametric and nonparametric data of the groups. Spearman's rho correlation test was used for correlation analysis. Significance was accepted if *P* < 0.05.

## 3. Results

The demographic data and the laboratory results of the entire study group are summarized in Table 1.

HOMA levels were not different among groups. However BMI was found higher in OH group than EH and controls as expected. SH group had also higher BMI than the control group.

TSH levels were higher in OH group than the other groups (*P* < 0.0001). SH patients also have higher TSH levels than the EH and control groups (*P* < 0.0001 and *P* < 0.0001). TSH levels were in normal limits in both control group and EH group; EH patients have slightly higher TSH values than the control group; however no statistical difference was found (*P* = 0.05) (Table 2).

FT3 and FT4 levels were in normal limits in both EH and control groups and no difference was found in these parameters (*P* = 0.13 and  *P* = 0.08, resp.) between two groups. Free T3 and FT4 levels were also in normal reference ranges in SH group. However, SH patients had lower FT4 and FT3 levels compared to EH (*P* < 0.0001 and *P* = 0.002, resp.) and control group (*P* < 0.0001 and *P* < 0.0001). FT3 and TF4 levels were significantly lower in OH group than the other groups (*P* < 0.0001 and *P* < 0.0001) as expected.

Total cholesterol, LDL-C, and TG levels were higher in total Hashimoto groups than the control group (*P* < 0.0001 in each). HDL-C was similar among groups (*P* = 0.27). OH group has higher Total-C, LDL-C, and TG levels than EH and control group as expected (*P* < 0.0001). OH patients also had higher Total-C (*P* = 0.004), TG (*P* = 0.01), and LDL levels (*P* = 0.008) compared to SH group. SH group had higher Total-C (*P* < 0.0001), LDL-C (*P* < 0.0001), and TG levels (*P* = 0.001) than the control group (Table 3). SH group also had higher Total-C (*P* = 0.02) levels than the EH group; however LDL-C (*P* = 0.08) and TG levels (*P* = 0.16) were similar to the EH group (Table 4). On the other hand EH group has higher Total-C (*P* = 0.04) and LDL-C (*P* = 0.01) levels but similar TG (*P* = 0.05) levels to the control group.

Lp(a) levels were higher in total Hashimoto group than the control group (*P* = 0.005). OH patients had significantly higher Lp(a) levels compared to control group (*P* < 0.0001); also patients with excess Lp(a) were more common in OH than the control group (*P* < 0.0001). OH patients have similar Lp(a) levels to SH patients (*P* = 0.39) and EH group (*P* = 0.15); however, Lp(a) excess was more common in OH than the EH (*P* = 0.01) group. Although Lp(a) levels were not different between OH and SH (*P* = 0.86) group, excess Lp(a) was also similar (*P* = 0.32).

On the other hand SH group has similar Lp(a) levels (*P* = 0.86) to EH group and the control group (*P* = 0.07). In SH group excess Lp(a) was also similar to the EH group (*P* = 0.1). However, SH group has more Lp(a) excess than the control group (*P* = 0.001). Moreover EH patients also have similar Lp(a) levels to control group (*P* = 0.08), but in EH group excess Lp(a) was more common than in the control group (*P* = 0.03).

Lp(a) was positively correlated with Total-C (*P* < 0.0001, *r* = 0.41), LDL-C (*P* < 0.0001, *r* = 0.42), TG (*P* = 0.001, *r* = 0.22), and TSH (*P* = 0.003, *r* = 0.20) and also negatively correlated with FT4 (*P* = 0.003, *r* = −0.20). TSH was positively correlated with Total-C (*P* < 0.0001, *r* = 0.40), LDL-C (*P* < 0.0001,  *r* = 0.38), and TG (*P* = 0.005,  *r* = 0.19). FT4 was negatively correlated with Total-C (*P* < 0.0001,  *r* = −0.38), LDL-C (*P* < 0.0001,  *P* = 0.38), and TG (*P* = 0.007  *r* = −0.18). However, FT3 was negatively correlated with Total-C (*P* < 0.0001,  *r* = 0.29) and LDL-C (*P* = 0.0001,  *r* = 0.29), but no correlation was found with TG (*P* = 0.138) and Lp(a) (*P* = 0.058). There was no correlation between Lp(a) levels and thyroid autoantibodies ((*P* = 0.054, *r* = 0.19) for anti-TPO and for (*P* = 0.09,  *r* = 0.23) anti-TG).

## 4. Discussion

This cross-sectional study shows increased Total-C and LDL-C, TG and Lp(a) levels in Hashimoto cohort compared with controls. Lp(a) was increased only in OH patients, although hyperlipoprotein(a)emia was more frequent in even euthyroid Hashimoto patients.

Hashimoto's thyroiditis (chronic autoimmune thyroiditis) is the most common cause of hypothyroidism in iodine-sufficient areas of the world. Thyroid failure is seen in up to 10 percent of the population and its prevalence increases with age [4].

In hypothyroidism major cardiovascular changes occur. Decreased cardiovascular output and cardiac contractibility, increased peripheral vascular resistance, and reduced heart rate result in cardiovascular dysfunction. There are also significant changes in modifiable atherosclerotic risk factors such as dyslipidemia [8, 9].

Dyslipidemia is common in hypothyroidism. Hypothyroid patients have increased levels of Total-C and LDL-C [10]. This is due to decreased LDL receptors' activity, resulting in decreased catabolism of LDL and IDL [11]. Moreover, a decrease in lipoprotein lipase (LPL) activity is found in overt hypothyroidism which decreases the catabolism of TG rich lipoproteins; therefore OH patients showed to have increased TG levels [1, 11, 12]. In our cohort OH patients had increased Total-C, LDL-C, and TG levels compared to controls, in accordance with the literature. Also in SH patients reported to have increased Total-C and LDL-C [13–15]. In addition some studies have shown that, in SH, dyslipidemia may also be accompanied with increased TG levels [16, 17]. In our study, Total-C, LDL-C, and TG levels were higher in the SH than in the control group.

Interestingly, in our cohort EH patients also have increased LDL-C, Total-C, and TG levels compared to the control group. TSH levels in our EH patients were not different from the control group. However, most of EH patients' (35 of 56 patients; 62%) TSH values were in high-normal range (2–4 ng/mL). It has been recently reported that TSH levels in the upper limits of the reference range, in patients with coronary heart disease, had adverse effect on lipid profile [18]. Michalopoulou et al. reported increased Total-C and LDL-C levels in subjects with high-normal TSH levels but with positive antithyroid antibodies [19]. Also in a prospective population based study it has been shown that high TSH levels within the reference range may be associated with adverse serum lipids. TSH levels may covary with blood pressure and lipid levels among people with apparently normal thyroid function [20]. Similarly a recent large cohort study showed that TSH in the upper limit of the reference range (above 2.5 mIU/L) was associated with higher Total-C [21]. This phenomenon was supported by the HUNT study [20]. Also recently reported studies showed that autoimmunity may have some effect on dyslipidemia independent of the thyroid function [22]. In our cohort, the increased Total-C and LDL-C levels in EH patients imply that autoimmunity may have some adverse effect on lipid profile independent of the TSH value.

Lp(a) is a form of LDL-C in which Lp(a) and ApoB are covalently bound by a disulphide bridge; it may be particularly atherogenic and thrombogenic [23]. Several studies have pointed out that high Lp(a) levels are associated with atherosclerotic disease, including myocardial infarction [23, 24]. It was shown that Lp(a) levels are increased in patients with OH and decrease after LT-4 treatment [25]. However, most studies to date have not found significantly elevated Lp(a) levels in patients with SH and have not demonstrated any effect of LT-4 treatment on serum Lp(a) concentrations [25–27]. It has been reported that euthyroid males and postmenopausal females with thyroid autoimmunity present with increased Lp(a), implying the role of thyroid autoimmunity in the Lp(a) metabolism [28]. In our study Lp(a) was increased in OH group, although Lp(a) levels were similar in SH, EH, and even in control group. However, Lp(a) excess, which we defined as Lp(a) > 30 mg/dL, was more frequent in OH group compared to other groups. Moreover the interesting point in this study, Lp(a) excess, was also more frequent in SC and even EH than the control group. According to this finding, we may speculate that autoimmunity may have an influence on the Lp(a) metabolism, even with the normal TSH levels.

In conclusion, this study shows that, in Hashimoto thyroiditis even in normal TSH values, dyslipidemia may occur and increased Total-C, TG, LDL-C, and Lp(a) excess may occur, which are the potential risk factors for atherosclerosis, considering the fact that these patients should be closely followed up in terms of cardiovascular events.

## Figures and Tables

**Table 1:** Demographic and laboratory results of the groups.

	Overt hypothyroid *n* = 48	Subclinical hypothyroid *n* = 50	Euthyroid Hashimoto *n* = 56	Control *n* = 50	*P*
Age (mean ± SD)	35.79 ± 7.28	36.80 ± 8.19	36.30 ± 8.11	35.42 ± 7.64	0.46

TSH (mIU/mL)	11.6 [IQR: 7.27–26.2]	7.25 [IQR: 5.94–8.61]	2.39 [IQR: 1.66–3.13]	1.88 [IQR: 1.46–2.5]	<0.0001

FT3 (ng/dL)	2.09 ± 0.37	2.99 ± 0.36	3.19 ± 0.2	3.32 ± 0.43	<0.0001

FT4 (ng/dL)	0.82 [IQR: 0.69–0.89]	1.08 [IQR: 1.02–1.18]	1.17 [IQR: 1.07–1.23]	1.20 [IQR: 1.13–1.34]	<0.0001

Total-C (mg/dL)	228.16 ± 38.91	204.5 ± 40.9	183.6 ± 31.5	176.4 ± 13.6	<0.0001

TG (mg/dL)	138 [IQR: 110–160]	115 [IQR: 82.7–133.2]	98.5 [IQR: 76.7–140.7]	81.50 [IQR: 69.7–110]	<0.0001

HDL-C (mg/dL)	57.10 ± 13.73	56.34 ± 12.06	56.69 ± 11.66	54.68 ± 10.96	0.274

LDL-C (mg/dL)	144.5 [IQR: 120–166]	134 [IQR: 110–143]	116 [IQR: 102–136.7]	108.5 [IQR: 97.75–117]	<0.0001

Lp(a) (mg/dL)	32 [IQR: 15–50.3]	17.55 [IQR: 12.8–53.5]	17.85 [IQR: 15.5–38.20]	17.45 [IQR: 10.95–22.0]	0.003

Excess Lp(a) percent (%)	*N*: 25 (52%)	*N*: 21 (42%)	*N* = 16 (28%)	*N*: 6 (12%)	<0.0001

BMI (kg/m^2^)	26.64 ± 2.02	26.36 ± 2.22	25.58 ± 2.72	24.9 ± 2.52	<0.0001

HOMA	1.76 ± 0.54	1.68 ± 0.58	1.73 ± 0.55	1.59 ± 0.62	0.22

Normally distributed values are indicated as mean ± SD; nonnormaly distributed values are indicated as median and interquartile ranges.

TSH: thyroid stimulating hormone, FT4: free thyroxin, Total-C: total cholesterol, TG: triglyceride, HDL: high density lipoprotein, LDL: low density lipoprotein, Lp(a): lipoprotein(a), and BMI: body mass index; HOMA for insulin resistance index.

*P* values are the differences of total Hashimoto groups and control group.

**Table 2:** Demographic and laboratory results of the euthyroid Hashimoto patients and the control group.

	Euthyroid Hashimoto *n* = 56	Control *n* = 50	*P*
Age (mean ± SD)	36.3 ± 8.11	35.42 ± 7.64	0.49

TSH (mIU/mL)	2.39 [IQR: 1.66–3.13]	1.88 [IQR: 1.46–2.50]	0.06

FT3 (ng/dL)	3.19 ± 0.2	3.32 ± 0.43	0.13

FT4 (ng/dL)	1.17 [IQR: 1.07–1.23]	1.20 [IQR: 1.13–1.34]	0.08

Total-C (mg/dL)	183.6 ± 31.5	176.4 ± 13.6	0.04

TG (mg/dL)	98.5 [IQR: 76.7–140.7]	81.50 [IQR: 69.7–110.75]	0.05

HDL-C (mg/dL)	56.69 ± 11.66	54.68 ± 10.96	0.32

LDL-C (mg/dL)	116 [IQR: 102–136.7]	108.5 [IQR: 97.75–117]	0.01

Lp(a) (mg/dL)	17.85 [IQR: 15.5–38.20]	17.45 [IQR: 10.95–22.02]	0.94

Excess Lp(a) percent (%)	*N* = 16 (28%)	*N*: 6 (12%)	0.03

BMI (kg/m^2^)	25.58 ± 2.72	24.9 ± 2.52	0.19

HOMA	1.73 ± 0.55	1.59 ± 0.62	0.22

Normally distributed values are indicated as mean ± SD; nonnormaly distributed values are indicated as median and interquartile ranges.

TSH: thyroid stimulating hormone, FT4: free thyroxin, Total-C: total cholesterol, TG: triglyceride, HDL: high density lipoprotein, LDL: low density lipoprotein, Lp(a): lipoprotein(a), and BMI: body mass index; HOMA for insulin resistance index.

*P* values are the differences of euthyroid Hashimoto group and control group.

**Table 3:** Demographic and laboratory results of the subclinical hypothyroid and the control group.

	Subclinical hypothyroid *n* = 50	Control *n* = 50	*P*
Age (mean ± SD)	36.80 ± 8.19	35.42 ± 7.64	0.31

TSH (mIU/mL)	7.25 [IQR: 5.94–8.61]	1.88 [IQR: 1.46–2.50]	<0.0001

FT3 (ng/dL)	2.99 ± 0.36	3.32 ± 0.43	<0.0001

FT4 (ng/dL)	1.08 [IQR: 1.02–1.18]	1.20 [IQR: 1.13–1.34]	<0.0001

Total-C (mg/dL)	204.5 ± 40.9	176.4 ± 13.6	<0.0001

TG (mg/dL)	115 [IQR: 82.7–133.2]	81.50 [IQR: 69.7–110.75]	0.001

HDL-C (mg/dL)	56.34 ± 12.06	54.68 ± 10.96	0.51

LDL-C (mg/dL)	134 [IQR: 110–143]	108.5 [IQR: 97.75–117]	<0.0001

Lp(a) (mg/dL)	17.55 [IQR: 12.8–53.5]	17.45 [IQR: 10.95–22.02]	0.07

Excess Lp(a) percent *N* (%)	*N*: 21 (42%)	*N*: 6 (12%)	0.001

BMI (kg/m^2^)	26.36 ± 2.22	24.9 ± 2.52	0.03

HOMA	1.68 ± 0.58	1.59 ± 0.62	0.43

Normally distributed values are indicated as mean ± SD; nonnormaly distributed values are indicated as median and interquartile ranges.

TSH: thyroid stimulating hormone, FT4: free thyroxin, Total-C: total cholesterol, TG: triglyceride, HDL: high density lipoprotein, LDL: low density lipoprotein, Lp(a): lipoprotein(a), and BMI: body mass index; HOMA for insulin resistance index.

*P* values are the differences of subclinical hypothyroid and the control group.

**Table 4:** Demographic and laboratory results of the subclinical hypothyroid and euthyroid Hashimoto patients.

	Subclinical hypothyroid *n* = 50	Euthyroid Hashimoto *n* = 56	*P*
Age (mean ± SD)	36.80 ± 8.19	36.3 ± 8.11	0.76

TSH (mIU/mL)	7.25 [IQR: 5.94–8.61]	2.39 [IQR: 1.66–3.13]	<0.0001

FT3 (ng/dL)	2.99 ± 0.36	3.19 ± 0.2	0.02

FT4 (ng/dL)	1.08 [IQR: 1.02–1.18]	1.17 [IQR: 1.07–1.23]	0.01

Total-C (mg/dL)	204.5 ± 40.9	183.6 ± 31.5	0.02

TG (mg/dL)	115 [IQR: 82.7–133.2]	98.5 [IQR: 76.7–140.7]	0.16

HDL-C (mg/dL)	56.34 ± 12.06	56.69 ± 11.66	0.67

LDL-C (mg/dL)	134 [IQR: 110–143]	116 [IQR: 102–136.7]	0.08

Lp(a) (mg/dL)	17.55 [IQR: 12.8–53.5]	17.85 [IQR: 15.5–38.20]	0.86

Excess Lp(a) percent *N*: (%)	*N*: 21 (42%)	*N* = 16 (28%)	0.15

BMI (kg/m^2^)	26.64 ± 2.02	23.58 ± 2.72	0.10

HOMA	1.68 ± 0.58	1.73 ± 0.55	0.69

Normally distributed values are indicated as mean ± SD; nonnormaly distributed values are indicated as median and interquartile ranges.

TSH: thyroid stimulating hormone, FT4: free thyroxin, Total-C: total cholesterol, TG: triglyceride, HDL: high density lipoprotein, LDL: low density lipoprotein, Lp(a): lipoprotein(a), and BMI: body mass index; HOMA for insulin resistance index.

*P* values are the differences of subclinical hypothyroid and euthyroid Hashimoto group.
